# The role of big data in financial technology toward financial inclusion

**DOI:** 10.3389/fdata.2024.1184444

**Published:** 2024-05-07

**Authors:** David Mhlanga

**Affiliations:** College of Business and Economics, University of Johannesburg, Johannesburg, South Africa

**Keywords:** big data, FinTech, benefits, financial inclusion, sustainable development

## Abstract

In the rapidly evolving landscape of financial technology (FinTech), big data stands as a cornerstone, driving significant transformations. This study delves into the pivotal role of big data in FinTech and its implications for financial inclusion. Employing a comprehensive literature review methodology, we analyze diverse sources including academic journals, industry reports, and online articles. Our findings illuminate how big data catalyzes the development of novel financial products and services, enhances risk management, and boosts operational efficiency, thereby fostering financial inclusion. Particularly, big data's capability to offer insightful customer behavior analytics is highlighted as a key driver for creating inclusive financial services. However, challenges such as data privacy and security, and the need for ethical algorithmic practices are also identified. This research contributes valuable insights for policymakers, regulators, and industry practitioners, suggesting a need for balanced regulatory frameworks to harness big data's potential ethically and responsibly. The outcomes of this study underscore the transformative power of big data in FinTech, indicating a pathway toward a more inclusive financial ecosystem.

## Introduction

In the past several years, there has been a meteoric rise in the usage of big data in the financial technology (fintech) business, and it is anticipated that this trend will continue to play an increasingly crucial role in the sector (Mhlanga, 2020; Mhlanga and Dunga, 2020; Johnson et al., 2021; Allen et al., 2022). The term “big data” refers to the extensive and intricate data sets that are produced by organizations, governments, and private citizens (George et al., 2014; Pappas et al., 2018). This information can come from a wide variety of sources, including financial transactions, social media, and sensor data, amongst others (George et al., 2014; Pappas et al., 2018). The capability of processing and analyzing this data in real-time has the potential to completely revolutionize how fintech business's function and engage with their clientele (Yang and Li, 2018; Mehrotra, 2019). The application of big data in the financial technology business can influence a variety of facets of the sector, such as decision-making, the creation of new products and services, risk management and compliance, and the development of new technologies. fintech companies can identify patterns and trends that can be used to make better decisions, such as predicting which customers are most likely to default on loans or identifying fraudulent activities, by analyzing large amounts of data in real-time. This allows the companies to make more accurate predictions.

The utilization of big data also allows for the creation of more personalized financial products and services, such as individualized investment portfolios and tailored insurance policies, for example (Cohen, 2018; Shirazi and Mohammadi, 2019). According to research, financial institutions may see an increase in revenue of 5%−15% if they implement customization strategies (Mariia et al., 2020; Abdulquadri et al., 2021; Chandra et al., 2022). In addition, the identification of possible risks and the development of measures to reduce such risks are two of the most important roles that big data plays in risk management and compliance. Also, big data is playing an important part in the development of new technologies such as blockchain and artificial intelligence, which may be utilized by fintech organizations to improve decision-making and automate operations. These new technologies can be powered by big data. In recent years, numerous studies, and articles (Awotunde et al., 2021; Eltweri et al., 2021; Meng et al., 2021; Wang R. et al., 2021) have addressed various aspects of the topic with the role that big data plays in financial technology (fintech). This has resulted in a significant amount of attention being paid to the topic. These studies have brought to light the potential advantages of big data for fintech organizations, including improved decision-making, product and service development, risk management and compliance, as well as the creation of new technologies.

However, they have also brought to light some of the difficulties and constraints associated with the use of big data in fintech, such as the need for specialized skills and infrastructure, as well as concerns around the privacy and security of data. The ability to make better decisions is one of the primary advantages that big data offers to fintech companies. Studies have shown that big data can be used to identify patterns and trends in financial data that can be used to make better decisions, such as predicting which customers are most likely to default on loans or identifying fraudulent activities. One example of this would be predicting which customers would be most likely to default on loans (Agarwal et al., 2020; Zhuo et al., 2020; Awotunde et al., 2021). Even though there is a growing corpus of research on the function that big data plays in fintech, there are still a great many areas that require additional investigation. For instance, there is a need for additional study on the practical uses of big data in the financial sector, such as how to efficiently deploy big data systems in financial institutions.

This study recognizes the extensive research on financial technology (FinTech) in the 2020s (Agarwal et al., 2020; Awotunde et al., 2021; Ozili, 2021). However, a distinct gap persists in understanding the specific role of big data in advancing financial inclusion. Unlike prior research that broadly examines FinTech's impact, this paper delves into how big data, as a core component of FinTech, facilitates financial inclusion. We explore various ways in which big data enables inclusive financial services, thereby offering a theoretical contribution that intersects the realms of big data and financial inclusivity. This research is essential due to the current scarcity of studies on the subject. The primary objective is to thoroughly understand the role of big data in the financial technology (fintech) sector, including its usage in business, its various impacts, and its prospects in addressing the financial inclusion of the unbanked. This article aims to offer insightful perspectives for scholars, decision-makers, and financial technology (fintech) companies who seek to understand the influence of big data on the fintech industry, particularly in tackling the issue of financial exclusion.

## Review of important literature

### Financial inclusion

Financial inclusion is a complex notion that encompasses the objective of guaranteeing universal access to a diverse array of inexpensive and suitable financial services and products offered by established financial institutions, for both individuals and enterprises (Mhlanga, 2020, 2022a). The primary objective is to integrate those who lack access to banking services or have limited access to such services into the conventional financial system. Financial inclusion refers to the act of granting individuals and businesses, particularly those who have been historically marginalized or excluded from the formal financial system, the opportunity to avail themselves of a diverse range of financial services and products. These offerings are crucial for effectively managing their financial affairs, attaining their economic objectives, and safeguarding against potential risks. The services possess characteristics that make them accessible, economical, easy, and customized to cater to the distinct requirements of various consumer segments.

Financial inclusion encompasses a diverse array of initiatives, such as the provision of fundamental banking services including savings accounts and checking accounts, as exemplified by basic banking services. As an illustration, a governmental entity may implement basic banking services that entail minimal or nonexistent charges, hence facilitating the accessibility and sustainability of bank accounts for those with limited financial resources. In addition, microcredit is among the range of services provided. Microcredit organizations provide financial services in the form of small loans to individuals engaged in entrepreneurial activities and small-scale business operations, who may face challenges in meeting the eligibility criteria for obtaining conventional bank loans. This enables individuals to initiate or broaden their entrepreneurial endeavors. The Grameen Bank, located in Bangladesh, is widely recognized as a prominent exemplar of a microcredit organization. Furthermore, digital payments are also present. Mobile money services, such as M-Pesa in Kenya, have significantly transformed the landscape of financial inclusion in numerous developing nations. Mobile phones provide individuals with the capability to transmit, receive, and save funds, hence obviating the necessity for conventional banking services.

Community-based financial institutions, such as credit unions and cooperatives, frequently cater to local communities by offering financial services to individuals who may lack access to traditional commercial banks. These financial organizations provide savings accounts, loans, and other services specifically designed to meet the requirements of the community. In addition, there exist government welfare programmes. Financial inclusion is a commonly employed strategy by governments to facilitate the direct transfer of welfare benefits and subsidies to the bank or mobile money accounts of recipients. An example of an effective programme is India's Direct Benefit Transfer (DBT) initiative, which aims to ensure the efficient delivery of social welfare payments to their intended beneficiaries. Financial education is an additional significant facet of financial inclusion. Financial inclusion includes the provision of educational and literacy initiatives aimed at enhancing individuals' comprehension of financial principles, proficient management of their finances, and the ability to make well-informed financial choices. This enables individuals to make prudent use of financial services. Insurance services play a crucial role in various contexts. Financial inclusion refers to the provision of access to various insurance products, such as health insurance, life insurance, and property insurance. Microinsurance programs provide insurance coverage to communities that are vulnerable to natural catastrophes in places that are prone to such events.

Furthermore, it encompasses the provision of investment opportunities, including stocks, bonds, and mutual funds. The advent of online investing platforms and robo-advisors has facilitated accessibility to investment opportunities, enabling individuals with limited wealth to engage in financial activities. Another crucial factor to consider is the availability of credit. Financial inclusion plays a pivotal role in facilitating individuals and small enterprises to avail credit facilities, hence enabling them to secure loans for diverse objectives. The proliferation of online peer-to-peer lending platforms has facilitated enhanced credit accessibility for borrowers who may not satisfy conventional lending prerequisites. Lastly, there is the provision of access to rural and distant areas. Financial inclusion projects frequently aim to expand access to financial services in rural and remote regions that may have limited traditional banking infrastructure. One illustration of this phenomenon is the provision of financial services in rural areas using mobile banking agents, thereby extending access to previously underserved communities. In essence, financial inclusion extends beyond mere possession of a bank account, encompassing a diverse array of financial services and products that enable individuals and enterprises to actively engage in the formal financial system. The initiative facilitates economic growth, mitigates poverty, and fosters financial resilience by enabling everyone access to opportunities for enhancing one's financial welfare.

### The meaning of fintech

The word “fintech,” which stands for “financial technology,” refers to the application of various forms of technology to enhance and automate financial services (Ferdiana and Darma, 2019; Mhlanga, 2020, 2022b; Firmansyah et al., 2023). This can cover a wide variety of applications, ranging from investment management and insurance to mobile banking and digital payments (Lee and Shin, 2018; Wewege et al., 2020). Fintech companies leverage technology to develop brand-new financial goods and services, as well as to enhance and simplify the delivery of existing products and services. Companies can also utilize technology to acquire data, analyze it, and use it to their advantage to develop new technologies and make better financial decisions. The financial technology business is expanding at a rapid rate and has the potential to challenge the traditional financial services industry by making traditional financial services more effective, accessible, and inexpensive.

### Big data

Big data refers to the collection, storage, and analysis of extremely large and complex sets of information (Lv et al., 2019; Aho and Duffield, 2020; Yang et al., 2020). This data can come from a variety of sources, including social media, sensors, and transactional systems, and it can be structured, semi-structured, or unstructured. The volume, velocity, and variety of big data make it difficult to process using traditional data processing techniques. One key aspect of big data is its scale. The amount of data being generated is rapidly increasing, and organizations are struggling to keep up with the sheer volume of information (Vlassenroot et al., 2021; Rehman et al., 2022). In addition to the volume of data, big data also has high velocity, meaning that it is generated and must be processed in real time. This is particularly important in industries such as finance and healthcare, where timely decisions are critical. Another important aspect of big data is its variety. Big data can come in many different forms, including text, images, videos, and audio. This makes it difficult to process using traditional data processing methods, which are often designed to handle only specific types of data. To handle the scale, velocity, and variety of big data, organizations are turning to new technologies and techniques, such as Hadoop, Spark, and NoSQL databases (Storey and Song, 2017; Oussous et al., 2018; Venkatesh et al., 2019). These technologies are designed to handle large, complex sets of data and make it possible to perform advanced analytics, such as machine learning and natural language processing. Despite the many benefits of big data, several challenges need to be addressed. One major challenge is data privacy and security. With so much personal information being collected, it is essential to ensure that this data is protected from unauthorized access and breaches. In addition, organizations must also ensure that they are complying with relevant regulations, such as the General Data Protection Regulation (GDPR) in Europe (Goddard, 2017; Voigt and Von dem Bussche, 2017). Another challenge is the lack of skilled personnel. With the rapid growth of big data, there is a shortage of professionals who are trained to work with these technologies and techniques. This is a significant barrier for organizations that want to take advantage of big data but lack the necessary expertise. In short, big data refers to the collection, storage, and analysis of extremely large and complex sets of information. It poses a challenge for traditional data processing techniques and requires new technologies and techniques, such as Hadoop, Spark, and NoSQL databases to handle its scale, velocity, and variety. It offers many benefits but also poses challenges such as data privacy and security, lack of skilled personnel and compliance with regulations.

### Crowdfunding

Crowdfunding is a method of raising capital that leverages the collective effort of friends, family, customers, and individual investors. This approach is primarily conducted online via social media and crowdfunding platforms, effectively tapping into the networks of a large pool of individuals for greater reach and exposure (Dresner, 2014; Zhao et al., 2022). At the heart of crowdfunding is the project initiator who proposes the idea or project to be funded, backed by individuals or groups who support the idea, and a moderating organization the platform that brings these parties together to launch the idea. There are several types of crowdfunding. Rewards-based crowdfunding involves backers receiving a tangible item or service in return for their funds. In donation-based crowdfunding, people donate to a cause, project, or event with no expectation of receiving anything in return (Gupta, 2018; Oh et al., 2023). Equity crowdfunding allows backers to receive shares of a company, usually in its early stages, in exchange for the money pledged. Debt crowdfunding, on the other hand, involves investors lending money to a company with the expectation that it will be paid back with interest. Crowdfunding platforms like Kickstarter, Indiegogo, GoFundMe, and others have emerged as popular means to solicit funds for a wide range of projects. This method offers several advantages. It provides access to a wide audience, enables the testing and validation of ideas, and offers an opportunity to engage and involve customers and fans in the development of a product or project. However, it also poses challenges. It requires a compelling pitch, carries the risk of not meeting funding goals, and presents the possibility of public failure. Crowdfunding represents a unique blend of technology, social networking, and finance, transforming traditional ways of funding projects and ventures.

### The Inclusion-Through-Technology theory

According to the Inclusion-Through-Technology theory, technological advancements, most notably the provision of digital financial services, have the potential to be utilized to broaden the scope of financial inclusion. This hypothesis proposes that digital financial services have the potential to help overcome barriers to access and reduce the costs of providing financial services, hence making them more available to underbanked populations. One of the most important things that technology can do to promote financial inclusion is to make it simpler for individuals to gain access to various forms of financial assistance (Loubere, 2017; Arner et al., 2020; Mhlanga, 2022a). For instance, mobile banking and digital wallets can provide users with the ability to access financial services from the convenience of their mobile devices, even in underserved or rural regions. Additionally, digital financial services can be used to reach underbanked populations that may not have access to traditional banking services, such as migrant workers or individuals with low incomes. This is possible because digital financial services can reach more people than traditional banking services do. One more way technology can help promote financial inclusion is by lowering the expenses that are associated with the provision of financial services. For instance, the cost of operating digital financial services can be lower than the cost of operating traditional brick-and-mortar branches, which can make these services more accessible to communities that are currently underserved. Additionally, the use of digital financial services can assist to cut down on the fees associated with financial transactions while also making it simpler for users to send and receive money. When compared to their analog counterparts, traditional financial services, and digital financial services offer a higher level of both security and transparency. For instance, with the use of the technology known as digital ledgers, digital transactions may be monitored and documented, which can contribute to the reduction of fraud and the enhancement of transparency. To summarize, the Inclusion-Through-Technology hypothesis proposes that technology has the potential to become a potent instrument in the promotion of financial inclusion. This can be accomplished by making financial services more accessible and inexpensive to underbanked people. Yet, it is essential to keep in mind that the successful implementation of digital financial services calls for a solid infrastructure in addition to a solid comprehension of the requirements and patterns of the population.

### Empirical literature review

In the past few years, there has been a significant increase in the amount of published material that examines the function of big data in financial technology (fintech). This is because researchers and practitioners have been trying to comprehend the influence that this technology has had on the sector. There have been several studies that have concentrated on the application of big data in particular aspects of fintech, such as risk management, product creation, and compliance. Other studies have investigated the more widespread effects that big data has had on the fintech business. The management of risks is one area of fintech that has benefited from substantial research into big data. Numerous studies have demonstrated that big data can be used to determine the existence of potential dangers and to formulate responses to those dangers. By way of illustration, a study conducted by Siddiqui et al. (2017) proved that big data may be utilized to detect fraudulent activity in real time by evaluating enormous amounts of data about financial transactions.

In another study, conducted by Wang et al. (2018), the researchers concluded that big data can be utilized to improve credit risk assessment by evaluating vast volumes of data about customers. This data can include social media and sensor data, among other types of data. It has also been demonstrated that big data has a substantial impact on the process of product development in the fintech industry. Numerous research has shown that big data may be utilized to generate more individualized financial products and services, such as individualized investment portfolios or personalized insurance policies. These examples are from the world of finance and business. For instance, He et al. (2023) demonstrated that big data may be utilized to build personalized financial solutions by evaluating vast volumes of data on clients. This data can include demographic information as well as data on financial transactions and data from social media. Further research (Ravi and Kamaruddin, 2017; Wen et al., 2020; Huang, 2021) demonstrated that by evaluating vast amounts of historical financial data with big data, it is possible to increase the accuracy of financial predictions. Big data has also been proven to have a substantial impact on compliance, which is another field. Research has concluded that big data can be utilized to improve regulatory compliance by identifying potential hazards and establishing ways to mitigate such risks. For instance, research conducted by Srivastava and Gopalkrishnan (2015) and Singh and Best (2019) found that big data can be utilized to improve compliance with anti-money laundering regulations by analyzing large amounts of data about financial transactions. This was accomplished using big data.

According to the findings of other research, such as that conducted by Altman et al. (2018) and Benjelloun and Lahcen (2019), big data can be utilized to better comply with data privacy requirements by evaluating vast amounts of data about customers. There have been several studies that have investigated the broader influence that big data has had on the fintech business. For instance, research conducted by Huang (2020) and Awotunde et al. (2021) discovered that big data may be utilized to increase the efficiency and efficacy of fintech organizations by evaluating massive volumes of data in real time. This was accomplished by using big data. Ivanchenko et al. (2019) and Holmlund et al. (2020) concluded that big data can be utilized to improve the customer experience by evaluating vast volumes of data on consumer behavior. This was discovered by both research teams. Big data can be used to improve the efficiency and effectiveness of fintech companies in areas such as risk management, product development, and compliance, according to the research that has been published on the topic of the role of big data in fintech. In a nutshell, this research has demonstrated that big data can be used. In addition, big data is playing a crucial part in the development of innovative technologies such as blockchain and artificial intelligence, both of which have the potential to be utilized by fintech organizations to enhance decision-making and automate a variety of procedures. However, additional study is required to have a better understanding of the larger influence that big data will have on the fintech industry, as well as the potential issues that could emerge in the future.

## Methods and data

### Research methodology

The study article's goal is to examine how big data contributes to financial inclusion in the world of fintech. The secondary data sources that this article is based on were examined. A few publications, a few policy reports, and reports from both national and international bodies are helpful to it. A systematic literature review is a research technique used to locate and compile previous studies that are pertinent to a certain research issue or area of study. It entails a thorough examination and evaluation of the selected studies after a thorough and organized search of the available literature. In a systematic review, a research question is developed, a protocol outlining the search strategy and inclusion criteria is created, a thorough search of pertinent databases and sources is carried out, studies that meet the inclusion criteria are chosen, their quality is evaluated, data are extracted, and conclusions are synthesized. A systematic literature review's objective is to offer a thorough and objective account of the existing research on a certain subject. It can be used to direct future research, fill up knowledge gaps, and inform policy. Because it is transparent, repeatable, and less biased than other research methods, a systematic literature review is regarded as a high-quality and rigorous research methodology.

### The systematic review process

**Figure 1:**
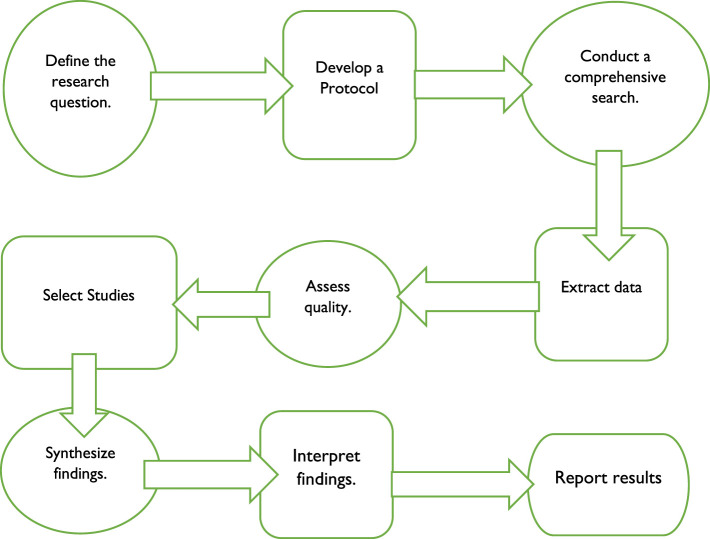
The systematic review process. Source: Author's Analysis.

The systematic review process involves several steps as shown in Figure 1.

**Figure 2:**
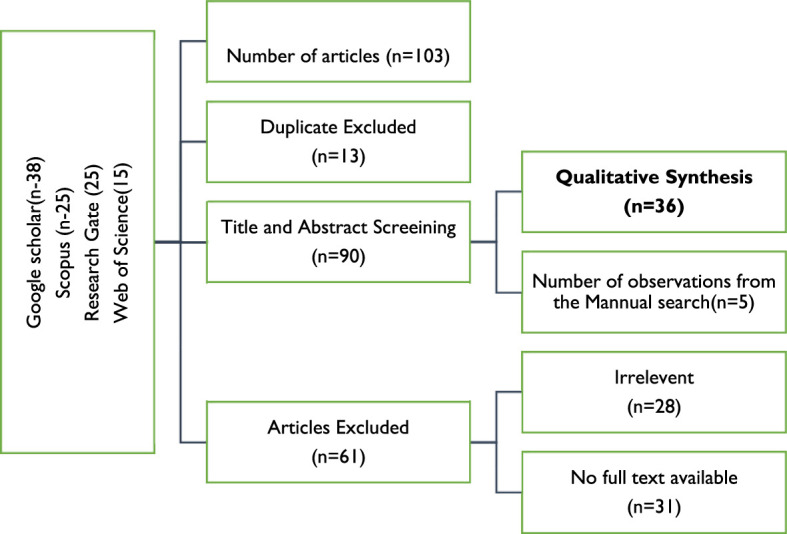
Flow diagram of studies' screening and selection. Source: Author's Analysis.

There are various steps in the systematic review process, some of which are depicted in Figure 1. The first stage is to precisely establish the research issue or topic that will serve as the review's compass. Creating a protocol that specifies the search method and inclusion/exclusion standards for studies. Also, it describes how to evaluate the chosen studies' quality and get data from them. Thus, a thorough search to find all pertinent studies comes next. Several databases, search terms, and inclusion/exclusion criteria are all part of the search strategy. The next step is to choose studies, where papers are screened according to inclusion and exclusion criteria and their applicability to the study topic. The chosen studies are next evaluated for their quality according to predetermined standards. The purpose of the quality evaluation is to locate any potential biases in the studies. The alternative method entails first extracting data from the chosen research using a predetermined data extraction form and then synthesizing the results. Statistical or narrative techniques are used to combine the findings of the chosen studies. The interpretation of the results then comes. The research question and the caliber of the investigations are taken into account while interpreting the results. The last step is to report the findings. The findings are presented in a systematic review report that details the methodologies employed, the findings, and the review's limitations. The systematic review procedure is intended to minimize bias in the selection and synthesis of research while still being open, repeatable, and transparent. It is a demanding and drawn-out process that needs meticulous preparation and execution. Figure 2 depicts the flow diagram that outlines the systematic review procedure.

To get a solution to the query, we adopted the method of doing a thorough analysis of the pertinent prior study. This allowed us to find the answer. According to Tawfik et al. (2019), the first thing that has to be done to have a successful systematic literature review is a preliminary search to locate relevant publications. This is the first step that needs to be completed. This will ensure that the idea that was proposed is sound, save us from responding to queries that have previously been answered, and assure that we have sufficient articles for analyzing the suggestion. Figure 2 provides a summary of the many pieces of research that were taken into consideration during the screening and selection processes. Because of the possibility of bias and errors caused by humans, we strongly advise having a data-checking stage in the process. Evidence images are utilized in the process of comparing each item included within the container to the entry that corresponds to it on an extraction sheet. The following are brief descriptions of various articles that were consulted throughout the research.

Table 1 provides an overview of the papers that were selected for inclusion in the study. The table only represents a small selection of the overall body of work that was considered for the research project.

**Table 1:** Selected articles consulted in the study.

**Study**	**Focus**	**Year**
Nobanee et al.	Big data applications the banking sector: a bibliometric analysis approach. *Sage Open* 11:21582440211067234	2021
Ennouri and Mezghani	Big data management in the era of FinTech: insights from a literature review. *Influence of FinTech on Management Transformation*, 102–120	2021
Firmansyah et al.	Factors affecting fintech adoption: a systematic literature review. *FinTech* 2, 21–33	2023
Weichert	The future of payments: how FinTech players are accelerating customer-driven innovation in financial services. *J. Payments Strategy Syst*. 11, 23–33	2017
Mhlanga	Industry 4.0 in finance: the impact of artificial intelligence (AI) on digital financial inclusion. *Int. J. Financ. Stud*. 8:45	2020
Zhuo et al.	How to integrate financial big data and fintech in a real application in banks: a case of the modelling of asset allocation for products based on data. *Information* 11:460	2020
Wang Y. et al.	Can fintech improve the efficiency of commercial banks?—an analysis based on big data. *Res. Int. Bus. Finance* 55:101338	2021

Source: Author's Analysis.

## Results and discussion

Big data's application in financial technology (fintech) has increased dramatically over the past several years, and it is anticipated to keep growing in significance. The role of big data in fintech, including its effects on decision-making, product and service creation, risk management and compliance, and the development of new technologies, will be thoroughly examined in this article. Big data is essential to financial technology (fintech) because it allows businesses to instantly evaluate vast amounts of financial data. As a result, they may spot patterns and trends that help them make smarter judgments, like figuring out which clients are most likely to default on loans or spotting fraud. Fintech businesses can develop more individualized financial goods and services using big data, such as customized investment portfolios or specialized insurance plans. Big data may also help with risk management, compliance, and the advancement of cutting-edge technology like blockchain and artificial intelligence.

**Figure 3:**
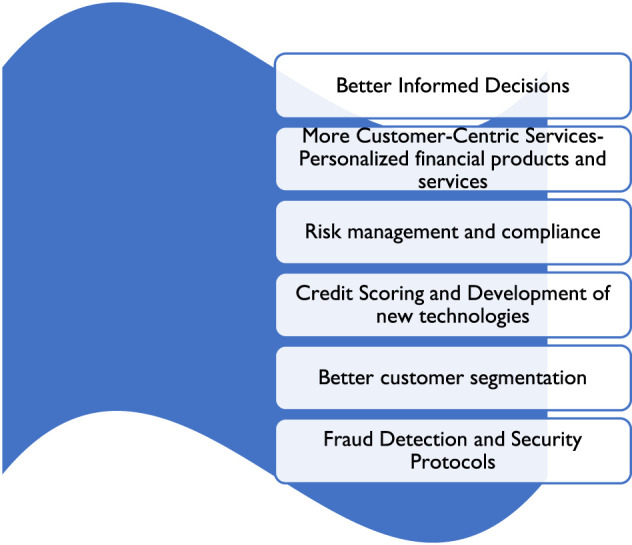
The use of big data in financial technology. Source: Author's Analysis.

The use of big data in financial technology, including making better-informed judgments, is illustrated in Figure 3. Other customer-focused services include, among others as listed above, risk management, compliance, and personalized financial products and services. Each of the contributions that big data has made to financial technology will be thoroughly explored in the sections that follow.

### Better informed decisions

For fintech organizations, big data has become a potent tool for making more informed decisions (Treleaven, 2015; Morales et al., 2022). Big data primarily enables fintech firms to instantly evaluate enormous amounts of financial data. This makes it possible for them to spot patterns and trends that may be used to guide their decision-making (Hassani et al., 2018; Polak et al., 2020; Roszkowska, 2021). Big data, for instance, can be used to forecast which clients are most likely to default on loans, enabling lenders to identify high-risk clients and create mitigation techniques for that risk (Huang et al., 2020; Sheng, 2021). Big data can be used to spot fraudulent behavior, such as shady account activity or suspect transactions. Fintech businesses may prevent losses and safeguard the financial information of their clients by promptly detecting and responding to suspected fraud by analyzing massive amounts of data in real time. The capacity to obtain a more in-depth understanding of customer behavior is another significant advantage of big data for fintech (Ennouri and Mezghani, 2021; Nobanee et al., 2021). Large volumes of data may be analyzed by fintech businesses to learn how customers use their goods and services, what their preferences are, and what influences their decisions (Hommel and Bican, 2020; Dehnert and Schumann, 2022). With the use of this data, fintech businesses may create goods and services that more effectively cater to the wants of their clients and cater their offerings to specific clients. Big data analytics, for instance, can assist fintech businesses in finding trends in consumer spending and transaction histories, which they can then utilize to provide individualized financial advice and investment suggestions. Big data can also assist fintech companies in better risk management.

Fintech businesses can spot possible hazards and take action to reduce them by analyzing vast amounts of data on market trends and client behavior. For instance, fintech businesses can employ big data analytics to track market trends and spot potential political or economic concerns that could influence their operations. Additionally, they can employ data analytics to evaluate potential borrowers' creditworthiness and make wiser loan and investing decisions (Serrano-Cinca et al., 2015; Kshetri, 2016). Big data can also assist finance companies to enhance their processes and raise productivity. Fintech organizations can pinpoint areas for improvement and put changes into place to optimize their processes and cut expenses by analyzing vast volumes of data on their business interactions and consumer interactions. Fintech businesses, for instance, can detect bottlenecks in their customer onboarding process using big data analytics and then simplify it to spend less time and resources to onboard new clients. To put it briefly, big data has become a potent tool for fintech organizations to use when making more educated judgments. Insights into consumer behavior, market trends, and risk management can be gained by analyzing enormous volumes of data by fintech businesses. These insights can be used to improve product design, identify, and stop fraud, better manage risk, and streamline operations.

### More customer-centric services- personalized financial products and services

Fintech firms can develop more individualized financial products and services thanks to big data (Melnychenko et al., 2020; Awotunde et al., 2021). Big data can be used, for instance, to assess a customer's financial background, income, and spending patterns to design personalized investment portfolios or specialized insurance policies (Cohen, 2018; Riikkinen et al., 2018). Fintech businesses may enhance revenue and improve client acquisition and retention by using big data to develop customized goods and services. Big data is playing a bigger role in the financial sector and has completely changed how fintech businesses run. Fintech businesses can employ significant insights into client behavior, tastes, and needs to be gained through the analysis of vast volumes of data to provide tailored financial products and services. These are a few ways big data might assist fintech businesses in providing customized financial goods and services. The second problem is that by using big data analytics, fintech organizations can divide their clientele into various groups according to their behavior, demographics, and preferences (Rizvi et al., 2018; Kovács et al., 2021). This enables fintech businesses to customize their goods and services for each group and make recommendations based on the needs of the customer. Fintech businesses, for instance, can utilize big data analytics to pinpoint the clients most likely to be interested in specific financial goods, such as loans or investment opportunities (Lee and Shin, 2018; Huttunen et al., 2019). Companies can then give these clients targeted marketing campaigns and provide tailored product suggestions based on their preferences and requirements. The other significant issue is that big data can assist fintech businesses in evaluating the risk of providing customized financial products and services to specific clients.

Fintech companies can determine the possibility of a consumer defaulting on a loan or other financial product by analyzing vast volumes of data on customer behavior, credit scores, and financial history. With the use of this data, loan conditions and interest rates can be tailored to the risk tolerance of each consumer. Making forecasts about consumer behavior and financial requirements is another application for big data analytics. Fintech businesses can forecast future financial demands and provide individualized suggestions for financial products and services by examining historical behavior and market patterns. For instance, based on prior purchasing habits and credit histories, fintech businesses can use predictive analytics to identify customers who are likely to require a loan soon. Based on each customer's unique financial requirements and risk tolerance, they can then offer them tailored loan terms and interest rates. Real-time insights into consumer behavior can also be obtained from big data, and these insights can be utilized to offer tailored financial services and products. Fintech firms can spot chances to provide customized financial products and services, such as investment opportunities or credit lines, by tracking consumer transactions in real time (Yang and Li, 2018; Awotunde et al., 2021). Fintech businesses, for instance, can use real-time data analytics to spot customers who are spending more money than usual on goods or services. They can then provide these clients with tailored investment possibilities or credit lines based on their unique financial requirements and preferences. For fintech organizations, big data has essentially become a significant tool for providing customized financial goods and services. Fintech businesses can customize their products and services to specific clients, assess risk factors, forecast future financial demands, and offer real-time insights into customer behavior by analyzing vast volumes of data on customer behavior, preferences, and wants. This can aid fintech businesses in boosting client happiness, revenue, and competitiveness in the financial sector.

### Risk management and compliance

Big data is essential for managing risks and complying with regulations. Fintech businesses can identify possible hazards and create methods to mitigate them by analyzing vast amounts of data (Anshari et al., 2021; Kijkasiwat, 2021; Wang Y. et al., 2021). Big data can be used, for instance, to study market movements and spot impending financial crises in advance. This can assist fintech companies in creating risk management plans that will lessen the negative effects of these crises on their operations. Big data can also be used to ensure compliance with legal obligations, such as know-your-customer (KYC) and anti-money laundering (AML) laws (Chen, 2020; Thommandru and Chakka, 2023). Fintech companies may make sure they are adhering to regulatory requirements and safeguarding the financial information of their consumers by using big data to discover and evaluate questionable transactions. Big data analytics can assist fintech businesses in developing predictive models that determine the potential of fraud, default, or other hazards related to a certain client or transaction. Fintech businesses can spot and manage possible hazards before they materialize by examining patterns in transactional data, social media feeds, and other sources. Fintech firms can use predictive analytics to identify and stop financial crimes like money laundering. Once more, big data analytics may be used by fintech companies to track transactions in real-time, spot suspicious activity, and take fast action to stop fraud. Fintech organizations can reduce the possibility of loss or harm by immediately identifying and addressing potential hazards with the use of real-time monitoring.

One further concern pertains to the utilization of big data by financial technology (fintech) firms for the purpose of monitoring their adherence to legal requirements. Fintech enterprises possess the capability to identify instances of non-compliance and implement measures to ensure adherence to their legal and regulatory responsibilities by conducting extensive data analysis (Truby et al., 2020; Fletcher et al., 2021). Fintech companies can employ big data analytics to conduct investigations on their clients (Awotunde et al., 2021; Meng et al., 2021). Fintech enterprises have the capability to identify potential hazards associated with a certain consumer or transaction through the analysis of publicly accessible data and social media streams. Fintech enterprises can ensure compliance with regulatory standards and prevent inadvertent facilitation of unlawful activities through the implementation of these measures. Fintech enterprises can enhance their understanding of client behavior, preferences, and requirements through the application of big data analytics. This can facilitate fintech enterprises in developing products and services that are tailored to meet the specific needs of their clientele, thereby enhancing client satisfaction and fostering long-term loyalty. Big data has the potential to support fintech companies in effectively managing risk and ensuring adherence to regulatory norms through the deployment of various capabilities like as real-time monitoring, predictive analytics, compliance monitoring, customer due diligence, and an improved customer experience. Fintech companies have the potential to gain a competitive advantage and create a strong reputation as a trustworthy provider of financial services through the effective use of big data.

### Credit scoring and development of new technologies

Fintech organizations can greatly benefit from big data analytics in the creation of new technologies and credit scoring (Onay and Öztürk, 2018; Hung et al., 2020). In this response, we'll outline how big data may be applied to credit scoring and how it can help fintech companies create new solutions. The practice of analyzing a borrower's creditworthiness to ascertain their chance of defaulting on a loan or other debt is known as credit scoring (Cheng et al., 2007; Roy and Shaw, 2021). The conventional approach to credit scoring is looking at a person's income, employment history, and credit history, among other things. Big data, on the other hand, can offer a more complete picture of a person's creditworthiness, enabling fintech companies to enhance their credit scoring algorithms. In the development of emerging technologies like blockchain and artificial intelligence, big data is crucial (AI).

The utilization of big data analysis in examining blockchain transactions can facilitate the identification of patterns and trends, hence enabling the improvement of safety and scalability within blockchain networks. The utilization of big data extends to the training of artificial intelligence (AI) models, namely machine learning algorithms. These models can then be employed by fintech organizations to optimize decision-making processes and streamline operations. The use of big data by fintech companies to acquire alternative data sources, such as social media feeds, mobile device usage, and other behavioral data, is another significant concern. Fintech companies can learn more precise information about a person's creditworthiness by examining various sources. Massive volumes of data can be analyzed by machine learning algorithms to find patterns and connections that conventional approaches would miss. Fintech businesses may increase the accuracy of their credit score and make better loan decisions by utilizing these algorithms. Once more, fintech firms can track changes in a borrower's financial situation in real-time and modify their credit score accordingly. This can lower the risk of default by enabling real-time risk detection and mitigation for fintech companies.

Big data can be used by fintech organizations to create new technologies that enhance their service offerings and expedite operations. These are a few ways big data can support the creation of new technology. Large amounts of data can be mined using big data to find trends and insights that can guide the creation of new technologies. This can assist fintech businesses in locating areas for innovation and creating fresh goods and services that satisfy consumer demand. To sum up, big data may greatly aid fintech companies in developing new technologies and improving credit scoring. Fintech companies may increase the accuracy of their credit scoring, create new goods and services, and optimize their operations by utilizing the power of big data, providing them with a competitive edge in the market.

### Better customer segmentation

According to Li et al. (2021) and Casas-Rosal et al. (2023), customer segmentation is the practice of breaking customers into groups based on shared traits like preferences, behavior, or demographics. Fintech companies may better identify client wants and adjust their products and services to match those needs by segmenting their customer base. Customer segmentation can benefit greatly from big data, which provides more thorough and specific information about customers (Russom, 2011; Grover et al., 2018). These are several ways big data can assist fintech businesses in more accurate consumer segmentation. Large volumes of data from numerous sources, including social media, transactional data, and other behavioral data, can be mined using big data to find patterns and correlations that might help with consumer segmentation. To evaluate customer data and create consumer categories based on behavior and preferences, fintech organizations can utilize advanced analytics techniques like machine learning and predictive analytics. Real-time data can be used by fintech companies to track changes in consumer behavior and modify their segmentation approach as necessary. For instance, fintech organizations can modify their segmentation strategy to better target a consumer if they start to notice new behaviors or preferences. By examining consumer behavior and preferences, big data may be used to tailor the customer experience. Fintech businesses can use this to better cater their goods and services to specific clients, increasing client happiness and loyalty. Fintech organizations can create targeted marketing strategies that are more likely to connect with client groups by segmenting their customer base. This can aid fintech businesses in increasing the efficacy of their marketing initiatives and boosting return on investment. Fintech businesses can better manage risk and lower the possibility of default or other financial losses by segmenting consumers based on risk criteria like creditworthiness. Big data can be used to categorize clients according to where they are physically located. This can assist fintech companies in better targeting regions with their goods and services. Fintech businesses, for instance, can provide goods and services that are suited to the demands of customers in particular areas or run location-specific promotions. Fintech firms can also find chances for cross-selling and up-selling by segmenting clients based on their previous activity. Fintech businesses can offer complementary products or services that may be of interest to a consumer who has previously purchased a specific good or service, for instance.

Fintech businesses may also assess the lifetime worth of each customer through the analysis of customer data, which enables them to prioritize different customer segments and allocate resources accordingly. For instance, fintech businesses may decide to place more emphasis on high-value clients to increase sales and profitability. In summary, big data can be tremendously helpful to fintech companies in terms of consumer segmentation. Fintech businesses may improve customer happiness and loyalty as well as their competitiveness in the market by utilizing the power of big data to better understand their consumers and personalize their products and services to match their demands.

### Fraud detection and security protocols

For fintech businesses, fraud is a key worry, and as more financial transactions shift to digital channels, the danger of fraud rises (Weichert, 2017; Giudici, 2018). Big data can be an effective tool for identifying and stopping fraud as well as creating strong security protocols. These are some ways that big data might assist fintech companies with security protocols and fraud detection. Big data analytics can be used to track financial transactions in real time and search for trends or anomalies that can point to fraud (Habeeb et al., 2019; Chu and Yong, 2021). Fintech organizations are now able to detect and stop fraud in real-time thanks to machine learning algorithms that can be trained to recognize suspicious patterns based on prior data. Once more, fintech firms can utilize big data to combine information from numerous sources, including social media, transactional data, and other behavioral data, to get a more thorough picture of client behavior. Fintech businesses can spot patterns and anomalies in this data that might be signs of fraud by analyzing it. Identity verification is the other concern. Using a variety of data sources, including transactional data, social media profiles, and biometric data, big data can be used to confirm the identity of clients. This can aid fintech businesses in preventing fraud and other crimes like identity theft. Fintech businesses can employ predictive modeling to forecast the possibility of fraud based on previous data using techniques like machine learning algorithms. This can assist fintech businesses in developing proper security processes and proactively identifying potential fraud concerns. Furthermore, possible is behavioral analysis. Big data can be used to study consumer behavior and spot irregularities that might point to fraud. For instance, it may be a symptom of fraud if a consumer suddenly starts engaging in transactions that are out of character for them. Big data can be used by fintech companies to track adherence to legal requirements like anti-money laundering (AML) laws. Fintech businesses can spot transactions that might be suspect and alert regulatory authorities by analyzing client data. Potential cybersecurity dangers and vulnerabilities can be found via big data. Fintech businesses can spot possible dangers and take the necessary precautions to prevent them by studying network traffic, logs, and other data sources. In conclusion, big data can be an effective tool for building strong security standards for fintech organizations as well as for detecting and preventing fraud. Fintech firms may better analyze client behavior, spot possible fraud risks, and create effective security protocols to guard against fraud and other cybersecurity dangers by utilizing the power of big data.

### The challenges associated with the role of big data in financial technology (fintech) toward financial inclusion

**Figure 4:**
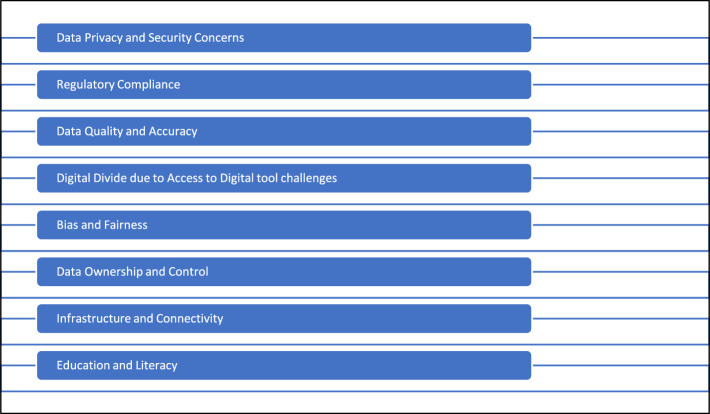
The challenges associated with the role of big data in financial technology (fintech) toward financial inclusion.

The incorporation of big data in the realm of financial technology (fintech) with the aim of achieving financial inclusion holds significant potential. However, this endeavor is accompanied by a range of obstacles that necessitate careful consideration and resolution to ensure its effective execution. Financial inclusion pertains to the provision of inexpensive and suitable financial services to communities that have historically been underserved and marginalized. Figure 4 outlines some of the challenges.

As depicted in Figure 4, there are a number of issues pertaining to the Role of Big Data in Financial Technology (fintech) in the context of achieving Financial Inclusion. One aspect pertains to issues over data privacy and security. The act of gathering and retaining substantial quantities of confidential financial information can give rise to notable concerns regarding privacy and security. The protection of consumer data from breaches and unauthorized access is of utmost importance. Another concern pertains to adherence to regulatory requirements. fintech enterprises encounter a multifaceted legal environment while managing client data, encompassing data protection legislation and financial regulatory frameworks. Adhering to these standards while harnessing the potential of big data can provide a formidable challenge. The quality and accuracy of data play a crucial role in the field of big data analytics. The presence of inaccurate or insufficient data has the potential to result in incorrect interpretations and judgements, hence potentially impeding the progress of financial inclusion initiatives.

The presence of a digital gap is a significant obstacle in including all users into fintech solutions driven by big data, as not everyone has equal access to digital devices or internet connectivity. The imperative of addressing the digital gap is crucial in order to achieve comprehensive financial inclusion for individuals across all socioeconomic backgrounds. Furthermore, it is important to consider the presence of bias and fairness issues in big data algorithms. These algorithms have the potential to perpetuate biases that exist in previous data, leading to outcomes that are unjust or discriminating. The imperative to establish inclusive financial services necessitates the assurance of fairness in algorithms and the mitigation of bias. In addition to the question of data ownership and control, there are further concerns to consider. The issue of ownership and control of data generated by fintech platforms can sometimes give rise to disputes and disagreements. It is imperative that customers are granted explicit ownership and control rights pertaining to their data. In addition to the aforementioned concerns, the matter of infrastructure and connectivity is also a significant issue. The availability of dependable and resilient internet connectivity is crucial for the utilization and accessibility of fintech services. Financial inclusion projects can face obstacles in remote or underprivileged communities due to infrastructure problems. Education and literacy pose significant challenges. The acquisition of financial literacy is of paramount importance for individuals in order to make well-informed decisions pertaining to their personal finances. fintech solutions sometimes presuppose a specific degree of digital and financial literacy, a condition that may not be universally present among all intended user groups. The resolution of these challenges requires a collaborative effort that involves fintech companies, regulatory agencies, and other pertinent stakeholders. The efficient use of big data for the equitable supply of financial services requires the attainment of an optimal balance between innovation, privacy, and security. This balance is essential for ensuring the wellbeing and autonomy of customers.

## Conclusion and policy recommendations

Concluding, this study underscores big data's transformative influence in fintech, significantly advancing financial inclusion. Our findings reveal that big data analytics in fintech has been pivotal in enabling more informed lending and investment decisions. Specifically, it has enhanced the accuracy of credit scoring models, allowing financial institutions to extend credit to previously underserved markets. Additionally, big data has fostered the development of tailored financial services for these groups, significantly reducing barriers to financial access. However, alongside these positive outcomes, our research also identifies challenges around data security, privacy, and potential algorithmic biases. These challenges highlight the critical need for robust ethical frameworks and regulatory compliance in the fintech sector. In particular, the growing reliance on automated decision-making systems demands greater transparency and accountability to prevent discriminatory practices. This research contributes to the evolving discourse on big data in fintech, offering insights for policymakers and industry practitioners. It underscores the need for a balanced approach that leverages the advantages of big data while mitigating its risks. Furthermore, our study emphasizes the importance of continued innovation in fintech, guided by ethical considerations and a commitment to inclusivity. Future fintech advancements should prioritize these considerations to harness big data's full potential responsibly. This study lays a foundation for subsequent empirical studies to explore the nuanced interplay between big data and financial inclusion, aiming for a more inclusive and equitable financial ecosystem. Ultimately, this research calls for a collaborative effort among stakeholders to realize the promise of big data in driving financial inclusion and economic empowerment.

## Author contributions

The author confirms being the sole contributor of this work and has approved it for publication.
